# Global prevalence of preterm birth among Pacific Islanders: A systematic review and meta-analysis

**DOI:** 10.1371/journal.pgph.0001000

**Published:** 2023-06-14

**Authors:** Bohao Wu, Veronika Shabanova, Kendall Arslanian, Kate Nyhan, Elizabeth Izampuye, Sarah Taylor, Bethel Muasau-Howard, Alec Ekeroma, Nicola L. Hawley

**Affiliations:** 1 Department of Chronic Disease Epidemiology, Yale School of Public Health, New Haven, CT, United States of America; 2 Department of Pediatrics, Yale School of Medicine, New Haven, CT, United States of America; 3 Department of Biostatistics, Yale School of Medicine, New Haven, CT, United States of America; 4 Department of Social and Behavioral Sciences, Yale School of Public Health, New Haven, CT, United States of America; 5 Harvey Cushing/John Hay Whitney Medical Library, Yale University, New Haven, CT, United States of America; 6 Department of Environmental Health Sciences, Yale School of Public Health, Yale University, New Haven, CT, United States of America; 7 Department of Obstetrics and Gynecology, Lyndon B Johnson Tropical Medical Center, Pago Pago, American Samoa; 8 National University of Samoa, Apia, Samoa; Federal University of Ceara, UNITED STATES

## Abstract

The epidemiology of preterm birth among Pacific Islanders is minimally understood. The purpose of this study was to estimate pooled prevalence of preterm birth among Pacific Islanders and to estimate their risk of preterm birth compared to White/European women. We searched MEDLINE, EMBASE, Web of Science Core Collection, Cochrane Library, CINAHL, Global Health, and two regional journals in March 2023. Observational studies were included if they reported preterm birth-related outcomes among Pacific Islanders. Random-effects models were used to estimate the pooled prevalence of preterm birth with 95% confidence interval (CI). Bayes meta-analysis was conducted to estimate pooled odds ratios (OR) with 95% highest posterior density intervals (HPDI). The Joanna Briggs Institute checklists were used for risk of bias assessment. We estimated preterm birth prevalence among Pacific Islanders in the United States (US, 11.8%, sample size [SS] = 209,930, 95% CI 10.8%-12.8%), the US-Affiliated Pacific Islands (USAPI, SS = 29,036, 6.7%, 95% CI 4.9%-9.0%), New Zealand (SS = 252,162, 7.7%, 95% CI 7.1%-8.3%), Australia (SS = 20,225, 6.1%, 95% CI 4.2%-8.7%), and Papua New Guinea (SS = 2,647, 7.0%, 95% CI 5.6%-8.8%). Pacific Islanders resident in the US were more likely to experience preterm birth compared to White women (OR = 1.45, 95% HPDI 1.32–1.58), but in New Zealand their risk was similar (OR = 1.00, 95% HPDI 0.83–1.16) to European women. Existing literature indicates that Pacific Islanders in the US had a higher prevalence of preterm birth and experienced health inequities. Learning from New Zealand’s culturally-sensitive approach to health care provision may provide a starting point for addressing disparities. The limited number of studies identified may contribute to higher risk of bias and the heterogeneity in our estimates; more data is needed to understand the true burden of preterm birth in the Pacific region.

## Introduction

In light of persistent disparities in perinatal health between minority and majority populations globally, greater attention is being paid to understanding unique risks that explain minority populations increased risk of preterm birth (live birth before 37 weeks gestation [1]). Despite efforts over the past several decades to intervene, preterm birth remains the leading cause of both neonatal and under five year death [2, 3]. In 2014, which is the most recent estimate from the World Health Organization, the global prevalence of preterm birth was 10.6% (uncertainty interval 9.0%-12.0%) [4].

Pacific Islanders are particularly underrepresented in perinatal health research, and little is known about the prevalence of preterm birth among this group. Data from the Pacific Islands themselves is sparse, both as a result of geographic isolation (~1000 islands across 300,000 square miles [5]) and nascent research infrastructure. Pacific Islander migrants are, however, among the fastest growing minority groups in the United States (US), New Zealand, and Australia. In the US, 1.2 million people identified as Native Hawaiian or Other Pacific Islanders (NHOPI) in 2010 [6]. In the 2018 census 24.6% of New Zealanders identified as Māori or Pacific Islander [7], and in Australia (2016), ~250,000 people reported Pacific Islander ethnicity [8]. While data from these settings should allow for ethnicity-specific examination of health outcomes, Pacific Islanders continue to be aggregated with other minority groups; in the US with Asian or Native Americans (or Pacific Islanders are omitted from analyses because of small sample size), and in Australia with Indigenous Australian groups.

Health challenges common among Pacific Islanders, such as, disproportionately high prevalence of obesity and obesity-related complications [5], may put them at a higher risk of preterm birth [5, 9]. Obesity is a significant risk factor for pre-eclampsia and pre-pregnancy diabetes [10, 11], which have been associated with indicated preterm births [12]. Endemic tropical illnesses may also increase risk [12, 13]: Papua New Guinea, for example, still has a high rate of malaria [14]. Furthermore, Pacific Islander migrants in developed countries may have limited access to social services [15–18] and reportedly experience discrimination [19], which may worsen their perinatal health outcomes.

To better understand the epidemiology of preterm birth among Pacific Islanders and the need for perinatal health intervention, this systematic review and meta-analysis aims to: (a) estimate the pooled prevalence of preterm birth among Pacific Islanders globally; and (b) identify whether Pacific Islanders were more likely to experience preterm birth compared to non-Hispanic White/European women.

## Materials and methods

### Protocol and registration

This review (PROSPERO ID: CRD42021283377, protocol [20]) followed Preferred Reporting Items for Systematic Reviews and Meta Analyses (PRISMA) [21] and Meta-analysis of Observational Studies in Epidemiology (MOOSE) reporting guidelines [22] and received Institutional Review Board exemption.

### Study population

Studies reporting outcomes of Pacific Islanders, including Native Hawaiians, residents in Micronesia, Melanesia, and Polynesia were eligible for inclusion. Countries/territories were selected based on the Pacific Island Countries [23] and previous studies [5, 15], including US-affiliated Pacific Islands (USAPI: American Samoa, Guam, Commonwealth of the Northern Mariana Islands [CNMI], Federated States of Micronesia, Republic of the Marshall Islands [RMI], and Palau), Kiribati, Nauru, Papua New Guinea, Solomon Islands, Fiji, New Caledonia, Vanuatu, Tonga, Tuvalu, Tokelau, Niue, French Polynesia, New Zealand (Māori, the indigenous Polynesian people of New Zealand), Samoa, and the Cook Islands. Studies from the US (Hawai’i and the contiguous US) and Australia (individuals from Ni-Vanuatu, Tahiti, and the Pitcairn islands; Aboriginal and Torre Strait Islanders were not included) were included, since there are large migrant populations in both countries.

### Information sources

We searched MEDLINE ALL (Ovid), EMBASE (Ovid), Web of Science Core Collection (as licensed at Yale [20]), Cochrane Library, CINAHL (EBSCOhost), and Global Health. The Pacific Journal of Reproductive Health and Pacific Health Dialog were searched manually since they are not well indexed in major bibliographic databases. Backwards and forwards citation chaining was searched by hand via Google Scholar. Reports from international, national, state-level, and territorial government agencies were searched manually.

### Search strategy

The search strategy was developed by the first author in consultation with all co-authors, including a medical librarian (KN). The search used two concepts: (1) Pacific Islanders and (2) preterm birth outcomes. Appropriate controlled vocabulary terms and keyword search terms were used (MEDLINE ALL example, **S1 Table**) and archived [24].

### Study selection

Peer-reviewed observational studies and agency reports published before March 17^th^ 2023 were included. Case reports and case-control studies were excluded due to inability to estimate prevalence. While doctoral theses were included, conference abstracts and master’s theses were not since final study outcomes may not have been available/reported.

Using Covidence data management software, each article was screened by two authors independently at the title-abstract and full-text screening stages. Authors met to reach consensus on inclusion and reasons for exclusion. Screening questions are presented in **S2 Table**. Studies using the same datasets with the same study period were examined for potential sample overlap. Where overlap was identified, only the study with the largest sample size was retained in analyses.

### Data extraction

Data extraction was completed by the first author and checked by the senior author. Extracted information included preterm birth-related outcomes (prevalence among Pacific Islander women and non-Hispanic White/European women), data source, publication date, data collection period, study country/setting, study design, gestational age (GA) measurement method, Pacific Islander ethnicity, and sample size.

For the prevalence estimate, if a study reported prevalence but not the absolute number of events, this number was calculated with *N*(Preterm Births) = P*N(total Pacific Islanders) and rounded to the next whole number, where N represented sample size and P represented prevalence of preterm birth. To compare risk among Pacific Islander and White/European women, the prevalence among the two groups and the modelling outcomes (odds radio [OR], modelling method, and adjusted confounders) were recorded. For studies reporting prevalence, OR were calculated with OR = ad/bc.

### Risk of bias assessment

The Joanna Briggs Institute (JBI) [25] checklist for prevalence studies [26] was used for the assessment in prevalence meta-analyses; the checklist for cross-sectional studies [27] was used for meta-analyses comparing risk. Two authors (BW and EI) completed appraisals and disagreements were discussed to reach consensus. Total scores represented the proportion of “checks” with 100% the maximum possible score. Egger’s test [28] (for prevalence meta-analyses) and weight-function models [29] (for Bayesian risk comparison meta-analyses) were used to assess publication bias.

### Synthesis of results

Stratified by study country/territory, we conducted prevalence meta-analyses of preterm birth among Pacific Islander women and comparison of preterm birth risk meta-analyses between Pacific Islander women and non-Hispanic White women in the US and New Zealand. As random-effects models (DerSimonian and Laird method) [30] consider the included studies to be a representative sample of possible articles on the research question of interest, these models were used for the pooled prevalence with 95% confidence intervals (CI). I^2^ is usually high in prevalence meta-analysis but may not indicate the data is inconsistent [31, 32], and it can be biased in small meta-analyses [33], so we reported tau with both the inverse variance (IV) method (weighting more to studies with a larger sample size) and the generalized linear mixed models (GLMM) method (weighting more to studies with smaller sample size) for the heterogeneity assessment. We compared the estimated tau^2^ [IV] to the within-study variances, with tau^2^[IV] larger than within-study variance indicating the weights of any two studies are approximately equal [34]. A large proportion of equal weights in a meta-analysis indicates the heterogeneity exists. Prediction intervals were presented in which future studies effects may fall based on present evidence. Subgroup analyses by Pacific Islander ethnicity were conducted to understand the between-study heterogeneity.

For the pooled risk comparison estimate, we used a Bayesian meta-analysis method [35] due to the relatively small number of studies identified by our search. Pooled ORs with 95% highest posterior density intervals (HPDI) were reported. We assumed no association, so we restricted the effect *ln*(*OR*) to normal prior centered at *ln*(*OR*)_*p*_ = 0 (no effect), and the prior standard deviation was restricted to *σ_p_* = 4; the priori expected heterogeneity was restricted to tau ≤ 0.98 with 95% probability as half-normal priori with scale 0.5. Tau with 95% HPDI were used for the heterogeneity assessment. Prediction intervals and subgroup analyses by ethnicity were also provided.

All analyses were performed using RStudio (RStudio, Inc., Boston, MA, USA). The R package **meta** [36] was used to conduct prevalence meta-analyses and to generate Forest plots and package **dmetar** [37] was used to perform Egger’s test for the corresponding publication bias assessment; package **bayesmeta** [38] was used to conduct Bayes meta-analyses, and package **RoBMA** [39] was used to perform the corresponding publication bias assessment (Bayes factor [BF]<3 indicating weak evidence[40]). R code is provided in **S1 Appendix**.

## Results

### Study selection

**Fig 1:**
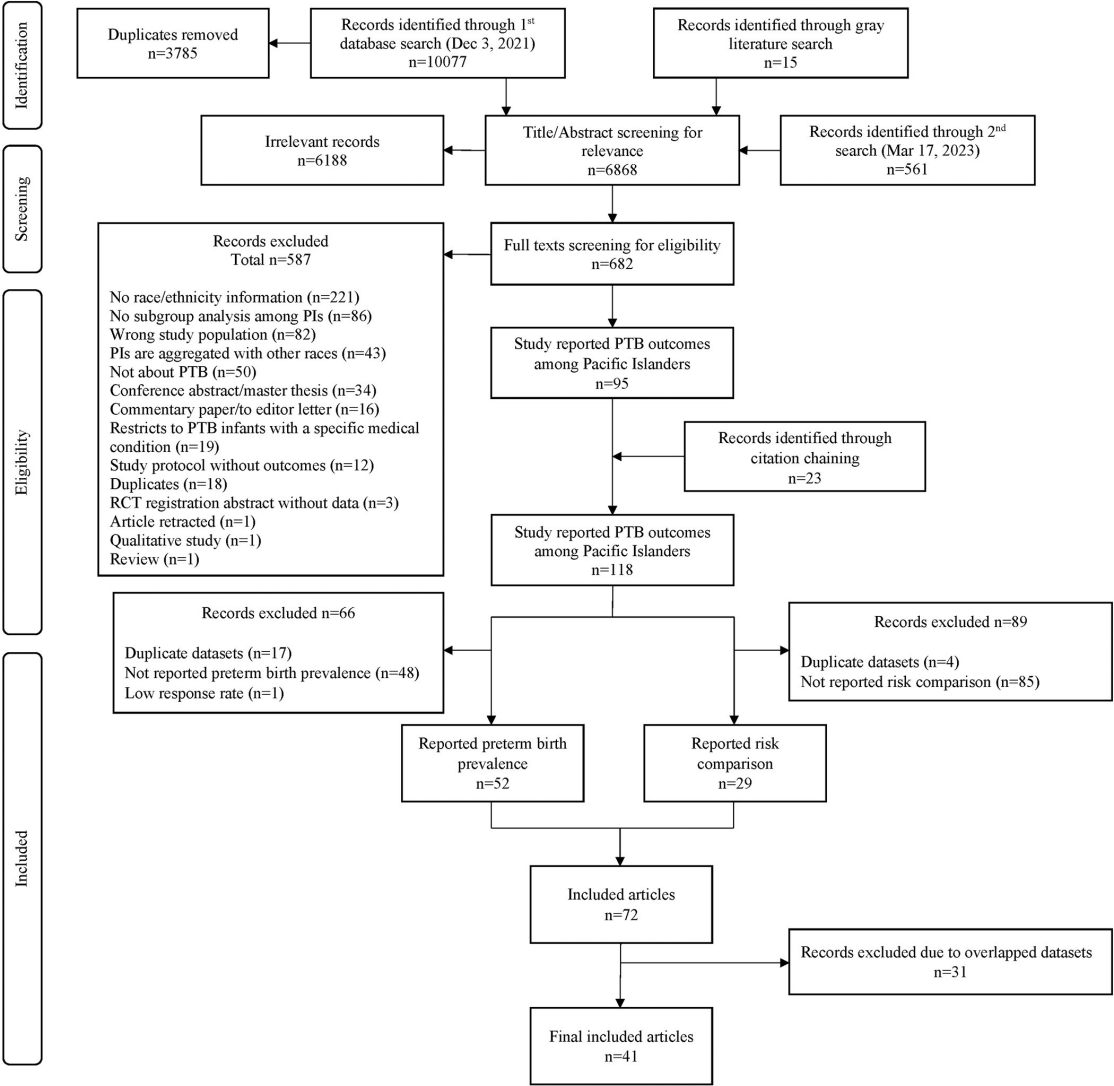
PRISMA flow diagram of study selection.

We identified 10,077 articles from six databases and 15 articles from gray literature on December 3^rd^ 2021, and updated our search (2nd search) in these six databases on March 17^th^ 2023 (**Fig 1**). After removing duplicates, title-abstract and full-text screening, and adding articles through citation chaining, we found 118 articles reporting preterm birth-related outcomes among Pacific Islanders among which 55 articles reported results related to our objectives. After removing studies with overlapping data (**S3 Table**), we included 33 articles that reported preterm birth prevalence, and 15 articles that compared risk between Pacific Islander and White/European women.

### Study characteristics

Of the 33 studies that reported prevalence of preterm birth among Pacific Islander women, 13 (39.4%) studies [41–53] were conducted in the US, 5 (15.2%) studies were conducted in the USAPI [54–58] (CNMI [57, 58], Palau [56], and RMI [54, 55]), 7 (21.2%) studies were from New Zealand [59–65], 2 (6.1%) studies were from Australia [66, 67], 4 (12.1%) studies [68–71] reported preterm birth prevalence in Papua New Guinea, 1 study was from Solomon Island [72], and 1 study was from Vanuatu [73] (**Table 1**). Of the 15 studies used for the risk comparison meta-analysis, 12 (80.0%) [41–48, 50, 53, 74, 75] were conducted in the US, and 3 (20.0%) were from New Zealand [76–78].

**Table 1:** Characteristics of included studies for preterm birth among Pacific Islanders.

Study	Data collection	Study design	State/Setting, Dataset	Pacific Islanders	GA measurement method	PTB definition
***US (N = 18*: *13 were used for prevalence estimate; 12 were used for risk comparison)***						
Crowell et al., 2007 [47] ^a^^,^ ^b^^,^ ^c^^,^ ^d^	1968–1994	Retrospective cohort study^e^	Hawaii State, birth record files	Hawaiian (n = 69,350) Samoan (n = 7,054) White (n = 108,981)	LMP	GA <37 weeks
Andrasfay et al., 2021 [48] ^a^^,^ ^b^ (G2 cohort)	1978–1995	Retrospective cohort study^e^	California, California birth records	Hawaiian/Pacific Islander (n = 1,577); White (n = 185,949)	LMP	GA <37 weeks
Korinek et al., 2021 [44] ^a^^,^ ^b^	1989–2015	Retrospective cohort study^e^	State of Utah, Utah Population Database	NHOPI (n = 10,438) White (n = 1,177,278)	Not reported	GA <37 weeks
Nembhard et al., 2019 [42] ^a, b, c, d^	1997–2013	Cross-sectional study	Arkansas, Vital record files	Marshallese (n = 2,488, missing value 270) Non-Hispanic White (n = 65,800)	Ultrasound (74%)	GA <37 weeks
Mathews et al., 2003 [79] ^c^	2001	Cross-sectional study^e^	US continent, National Vital Statistics Reports	Hawaiian (n = 6,411) White (n = 3,177,698)	LMP	GA <37 weeks
Hirai et al., 2013 [45] ^a, b, c, d^	2002–2009	Cross-sectional study^e^	Hawaii, Linked Infant Death Cohort Files	Native Hawaiian (n = 40,917) White (n = 33,683)	LMP	GA <37 weeks
Centers for Disease Control and Prevention et al., 2011 [52] ^a^	2003–2008	Descriptive study^e^	Washington State, King County, US government census	Hawaiians/Pacific Islanders (n = 2,200)	Not reported	GA <37 weeks
Wong et al., 2008 [80] ^c^	2003	Retrospective cohort study	US continent, National Natality Files	Samoan (n = 1,835) Guamanian (n = 30,353) Hawaiian (n = 1,302)	Not reported	GA <37 weeks
Schempf et al., 2010 [75] ^b, c, d^	2003–2005	Cross-sectional study^e^	California and Hawaii, birth certificates	Native Hawaiian (n = 16,805) Guamanian (n = 1,406) Marshallese (n = 938) Samoan (n = 4,820) Tongan (n = 1,594) White (n = 10,144)	CA: LMP; HA: clinical estimate	GA <37 weeks
Altman et al., 2019 [49] ^a, c^	2007–2012	Retrospective cohort study	California, birth records	Hawaiian (n = 756) Guamanian (n = 844) Samoan (n = 2,852) Other Pacific Islander (n = 5,422) More than one (n = 596)	Not reported	GA <37 weeks
Ratnasiri et al., 2018 [74] ^b^	2007–2016	Retrospective cohort study	California, Birth Statistical Master Files	N for races was not reported	Obstetric estimate	GA <37 weeks
Wartko et al., 2017 [41] ^a, b^	2008–2012	Retrospective cohort study	Washington State, King County, birth records	NHOPI (n = 1,853) White (n = 62,950)	Not reported	Not reported
Hawaii State Department of Health et al., 2019 [50] ^a, b, d^	2012–2015	Descriptive study^e^	Hawaii, Pregnancy Risk Assessment Monitoring System	Native Hawaiian (n = 5,050) Samoan (n = 300) Other Pacific Islander (n = 950) White (n = 4,400)	Clinical estimate	GA <37 weeks
Public Health Department, Seattle & King County et al., 2015 [51] ^a^	2013	Descriptive study^e^	Washington State, King County, birth certificate data	Pacific Islander (n not reported) White (n = 13,061)	Not reported	GA <37 weeks
Quan et al., 2021 [81] ^c^	2015–2017	Cross-sectional study^e^	National Natality File	NHOPI (n = 9,476)	Obstetric estimate	GA <37 weeks
Martin et al., 2019 [43] ^a, b^	2016–2018	Descriptive study^e^	US continent, Natality Data Files	NHOPI (n = 28,244) White (n = 6,005,206)	Obstetric estimate	GA <37 weeks
Hamilton et al., 2021 [46] ^a, b^	2019–2020	Descriptive study^e^	US continent, Natality Data Files	NHOPI (n = 19,382) White (n = 3,755,477)	Obstetric estimate	GA <37 weeks
Hamilton et al., 2022 [53] ^a, b^	2021	Descriptive study^e^	US continent, Natality Data Files	NHOPI (n = 9,517) White (n = 1,884,554)	Obstetric estimate	GA <37 weeks
***USAPI (N = 5)***						
Fox et al., 2005 [58] ^a^	1986–1996	Retrospective cohort study^e^	Commonwealth of the Northern Mariana Islands, hospital records	Women from Commonwealth of the Northern Mariana Islands (n = 10,756)	Determined by the birth attendant	GA <36 weeks
Dela Cruz et al., 2018 [57] ^a^	2007–2014	Retrospective cohort study	Commonwealth of the Northern Mariana Islands, hospital records	Chamorro/Carolinian (n = 2,799) Other Pacific Islander (n = 785)	Obstetric estimate	GA <37 weeks
Berger et al., 2016 [56] ^a^	2007–2013	Retrospective cohort study	Palau, hospital records	Palauan (n = 1,171)	LMP	GA <37 weeks
Ministry of Health in Republic of the Marshall Islands, 2010 [55] ^a^	2007–2010	Descriptive study^e^	Republic of the Marshall Islands, Vital Statistics Database	Women from Republic of the Marshall Islands (n = 6,116)	Not reported	Not reported
Ministry of Health in Republic of the Marshall Islands, 2016 [54] ^a^	2011–2016	Descriptive study^e^	Republic of the Marshall Islands, Vital Statistics Database	Women from Republic of the Marshall Islands (n = 7,515)	Not reported	GA <37 weeks
***New Zealand (N = 10*: *7 were used for prevalence estimate; 3 were used for risk comparison)***						
Cantwell et al., 1973 [63] ^a, c^	Not reported	Prospective cohort study	Hawkes Bay, hospital records	Maori (n = 276, missing value n = 21)	Not reported	Not reported
Wright et al., 1998 [59] ^a, c^	1987–1990	Cross-sectional study	Cot Death Study	Maori (n = 306) Pacific Islander (n = 154)	Clinical estimate	GA <37 weeks
Sadler et al., 2002 [61] ^a, c^	1992–1999	Retrospective cohort study^e^	Auckland, hospital records	Maori (n = 4,361) Pacific Islander (n = 8,197)	Not reported	GA <37 weeks
Lawton et al., 2016 [62] ^a, c^	1995–2009	Retrospective cohort study	Wellington region, hospital records	Maori (n = 6,960) Pacific Islander (n = 5,697) European (n = 33,386)	Not reported	GA <36 weeks
Craig et al., 2004 [76] ^b, d^	1996–2001	Retrospective cohort study^e^	Data file from New Zealand Health Information Service	Maori (n = 72,826, missing value 6,729, 9.2%) Pacific Islander (n = 32,713, missing value 3,515, 10.7%) European (n = 192,295, missing value 18,042, 9.4%)	Ultrasound	GA <37 weeks
Sundborn et al., 2011 [60] ^a, c^	2000	Cross-sectional study^e^	South Auckland, Pacific Island Families Study	Samoan (n = 627) Cook Island Maori (n = 230) Niuean (n = 57) Tongan (n = 278)	LMP	GA <37 weeks
Berry et al., 2018 [64] ^a, c^	2005–2015	Retrospective cohort study	The Department of Internal Affairs and the Ministry of Health’s National Minimum Dataset	Maori (n = 129,300) Pacific Islander (n = 72,435)	Ascertained by the obstetric care provider	GA <37 weeks
Parry et al., 2011 [78] ^b, d^	2007–2010	Prospective cohort study	Auckland, hospital records	Maori (n = 13,647) Pacific peoples (n = 6,625) European (n = 33,957)	LMP	GA <37 weeks
Edmonds et al., 2021 [77] ^b, d^	2010–2014	Retrospective cohort study	Kaupapa Maori research	Maori (n = 77,601) Pasifika (n = 33,106) European (n = 141,815)	Not reported	GA <37 weeks
Ministry of Health et al., 2019 [65] ^a^	2016	Descriptive study^e^	New Zealand Mortality Collection	Maori (n = 17,329) Pacific peoples (n = 5,976)	Not reported	GA <37 weeks
***Australia (N = 2)***						
Berman et al., 2021 [67] ^a^	2003–2016	Retrospective cohort study	Linked birth, hospital and death data from New South Wales	Oceania (n = 17,284)	Not reported	GA <37 weeks (Included 32–36 weeks only)
Mozooni et al., 2018 [66] ^a^	2005–2013	Retrospective cohort study	Dataset from Western Australia Data Linkage System	Maori (n = 2,941)	Not reported	GA <37 weeks
***Papua New Guinea (N = 4)***						
Garner et al., 1994 [69] ^a^	1984–1987	Cross-sectional study^e^	Wosera subdistrict, hospital records	Women from Papua New Guinea (n = 121)	Dubowitz assessment	GA <37 weeks
Allen et al., 1998 [68] ^a^	1994–1996	Prospective cohort study	Madang Province, hospital records	Women from Papua New Guinea (n = 987)	Ultrasound	GA <38 weeks
Senn et al., 2009 [70] ^a^	2007–2008	Cross-sectional study	Madang Province, hospital records	Women from Papua New Guinea (n = 310)	Not reported	GA <37 weeks
Unger et al., 2019 [71] ^a^	2009–2013	Prospective cohort study	Madang Province, hospital records	Women from Papua New Guinea (n = 1,229)	Ultrasound (65.5%); fetal biometry for the majority	GA <37 weeks
***Other Pacific Island countries (N = 2)***						
Cafaro et al., 2015 [72] ^f^	2011–2013	Retrospective cohort study	Records from KiraKira Hospital, Makira-Ulawa Province, Solomon Island	Solomon Islander (n = 1,233)	Not reported	GA<37 weeks
Therrien et al., 2021 [73] ^f^	2016	Cross-sectional study^e^	Records from Vila Central Hospital on Efate Island, Vanuatu.	Women from Vanuatu (n = 187)	Not reported	Not reported

GA, gestational age; LMP, last menstrual period; NHOPI, Native Hawaiian and Other Pacific Islanders; CA, California; HA, Hawaii

^a^ Labelled studies were included in preterm birth prevalence meta-analyses.

^b^ Labelled studies were included in the risk of preterm birth compared to White/European women meta-analysis.

^c^ Labelled studies were included in preterm birth prevalence subgroup meta-analysis.

^d^ Labelled studies were included in the risk of preterm birth compared to White/European women subgroup meta-analyses.

^e^ The study design was not reported and was decide by the first author’s judgement.

^f^ Labelled studies were not included in meat-analyses since there was only one study from each setting.

Almost half of the 41 total included studies [41, 44, 47, 49, 51–55, 61–63, 65–67, 70, 72, 73, 77, 79, 80] (43.9%) did not report the method used to estimate GA. Seven (17.1%) studies used last menstrual period (LMP) only [45, 47, 48, 56, 60, 78, 79] and seven (17.1%) used clinical/obstetric estimates [43, 46, 50, 57, 59, 74, 81]. In two (4.9%) studies [58, 64], GA was determined by the obstetric care provider at birth; two (4.9%) studies used ultrasound [68, 76] and one used Dubowitz assessment [69] (2.4%). The remaining three studies [42, 71, 75] (7.3%) used a combination of ultrasound, LMP, and/or clinical estimate. Most included studies defined preterm birth as occurring before 37 weeks, four (9.8%) [41, 54, 63, 73] did not report their GA threshold, two [58, 62] (4.9%) used <36 weeks, and one [68] (2.4%) used <38 weeks.

### Risk of bias

For the prevalence meta-analysis, risk of bias scores ranged from 55.6% to 100.0%, with higher scores indicating lower risk of bias (**S4** and **S5 Tables**). For the meta-analysis comparing risk, risk of bias scores ranged from 37.5% to 100.0%; confounders included in models and modelling methods were summarized for each study in **S6 Table**.

### Synthesis of results

#### Prevalence of preterm birth

**Fig 2:**
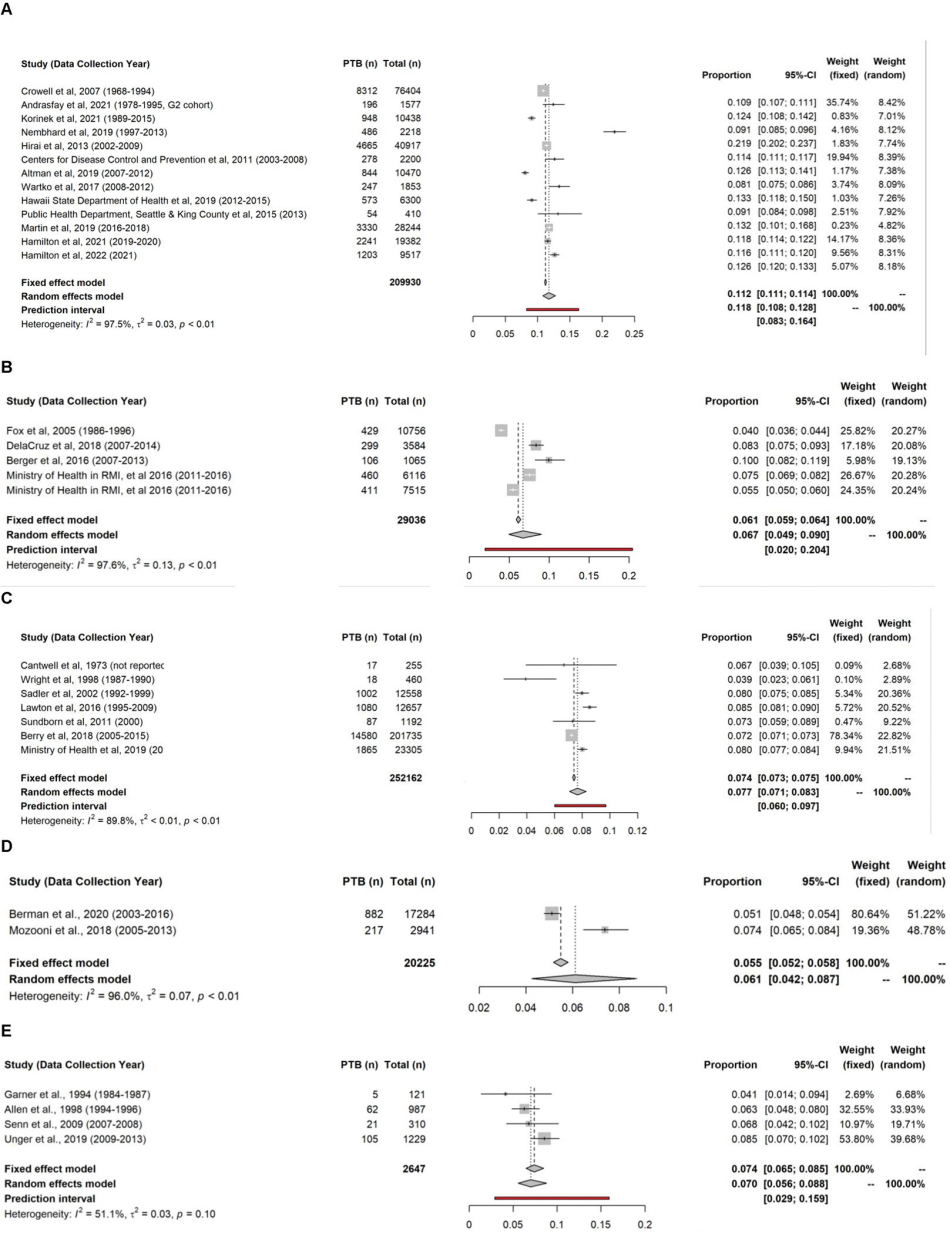
Forest plots of preterm birth prevalence among Pacific Islanders stratified by country/territory: (A) the US, (B) the USAPI, (C) New Zealand, (D) Australia, (E) Papua New Guinea^a^. ^a^ Prediction interval of Australia is not available.

In the US, the random-effects pooled prevalence of preterm birth among Pacific Islanders was 11.8% (95% CI 10.8%-12.8%; tau [IV] = 0.17, 95% CI 0.16–0.41; tau [GLMM] = 0.27), and the prediction interval ranged from 8.3% to 16.4% (**Fig 2A**). In subgroup analysis, the random-effects pooled prevalence of preterm birth among Marshallese (20.5%, 95% CI 17.7%-23.7%, tau[IV] = 0.12, tau[GLMM] = 0.06) was twice the pooled prevalence among the general Pacific Islander population in the US, and almost the same for other ethnicities (**S1 Fig**). The pooled prevalence was lower in the USAPI (6.7%, 95% CI 4.9%-9.0%, tau[IV] = 0.36, 95% CI 0.22–1.10; tau[GLMM] = 0.34), but the prevalence interval ranged from 2.0% to 20.4% (**Fig 2B**).

The random-effects pooled prevalence of preterm birth among Pacific Islanders overlapped in New Zealand, Australia, and Papua New Guinea (**Fig 2C–2E**). In New Zealand, the pooled prevalence was 7.7% (95% CI 7.1%-8.3%, tau[IV] = 0.09, 95% CI 0.06–0.36; tau[GLMM] = 0.08) and the prediction interval ranged from 6.0% to 9.7%. In subgroup analysis (**S2 Fig**), we found no obvious difference between the prevalence among Māori (8.4%, 95% CI 7.1%-9.8%) and other Pacific Islanders (7.5%, 95% CI 6.8%-8.3%). In Australia, the pooled prevalence was 6.1% (95% CI 4.2%-8.7%, tau[IV] = 0.27; tau[GLMM] = 0.19). The pooled prevalence of preterm birth in Papua New Guinea was 7.0% (95% CI 5.6%-8.8%, tau[IV] = 0.17, 95% CI 0–1.05; tau[GLMM] = 0.12), and the prediction interval ranged from 2.9% to 15.9%.

### Comparison of preterm birth risk between Pacific Islander and White/European women

**Fig 3:**
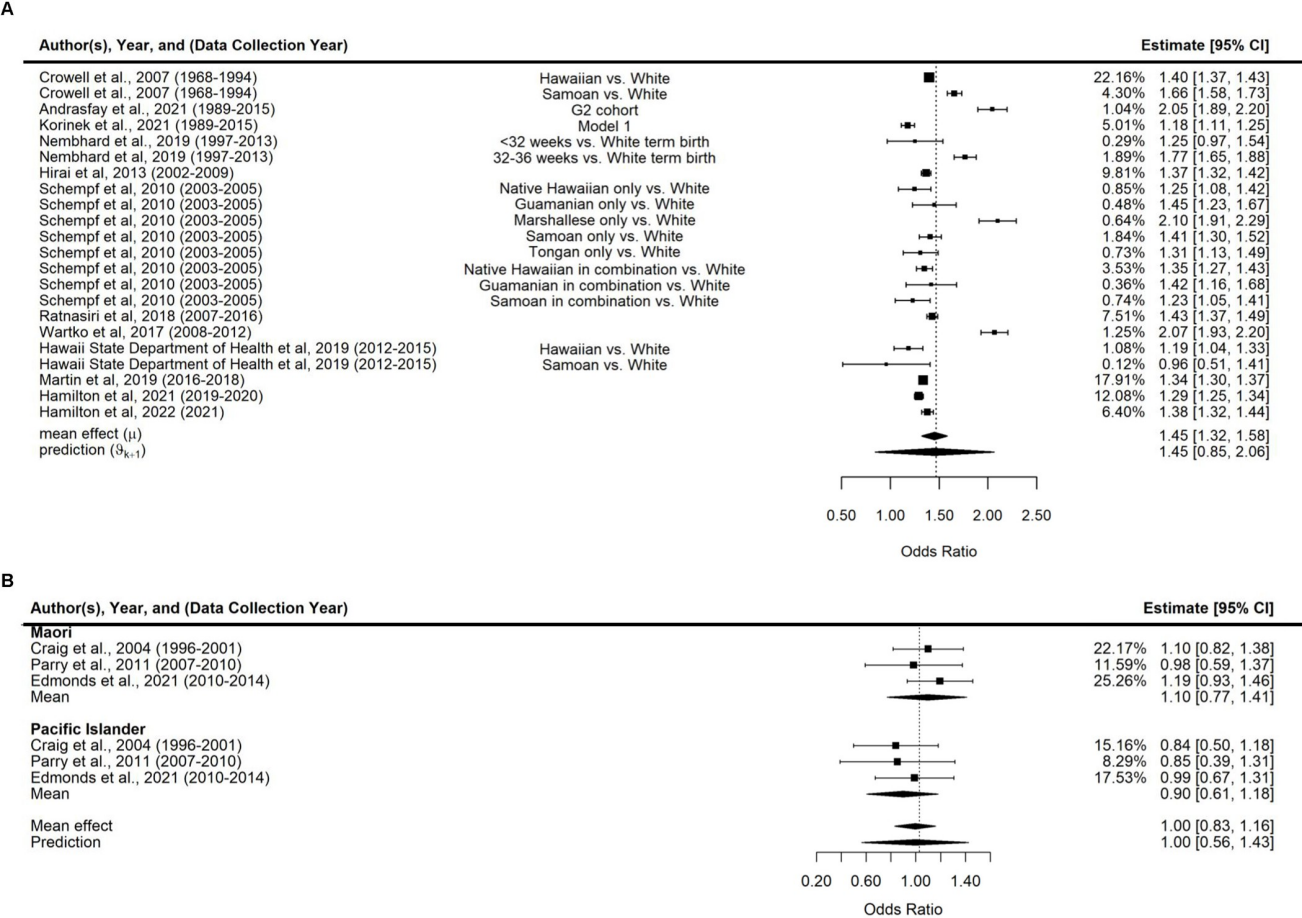
Forest plots of risk of preterm birth comparing Pacific Islander women to (A) non-Hispanic White women in the US and to (B) European women in New Zealand.

Based on 12 studies from the US, we estimated the pooled odds of preterm birth among Pacific Islander women to be 46% higher than among non-Hispanic White women (OR = 1.45, 95% HPDI 1.32–1.58, tau = 0.16, 95% HPDI 0.11–0.24 (**Fig 3A**). In subgroup analysis, Hawaiian (OR = 1.34, 95% HPDI 1.21–1.44, tau = 0.04, 95% HPDI 0.00–0.17), Marshallese (OR = 1.72, 95% HPDI 1.07–2.33, tau = 0.27, 95% HPDI 0–0.72), and Samoan (OR = 1.36, 95% HPDI 0.93–1.74, tau = 0.22, 95% HPDI 0.04–0.58) women were all more likely to experience preterm birth compared to non-Hispanic White women in the US (**S3 Fig**).

Using data from three studies from New Zealand we determined that the pooled odds of preterm birth were similar for Pacific Islander and European women (OR = 1.00, 95% HPDI 0.83–1.16, tau = 0.16, 95% HPDI 0.07–0.34, **Fig 3B**). Stratified by ethnicity subgroups, neither Māori (OR = 1.10, 95% HPDI 0.77–1.41, tau = 0.16, 95% HPDI 0.03–0.54) nor other Pacific Islanders (OR = 0.90, 95% HPDI 0.61–1.18, tau = 0.14, 95% HPDI 0.02–0.50) had higher odds of preterm birth compared to European women.

### Heterogeneity assessment

Checking the proportion of the studies with a smaller variance than the tau^2^[IV] suggests that no heterogeneity existed in the prevalence meta-analysis in the US (0%) and New Zealand (0%), however, meta-analyses conducted for the USAPI (100.0%), Australia (100.0%) and Papua New Guinea (100.0%) all showed strong evidence of heterogeneity. When using the same method to check the proportion of the studies with a smaller variance of log scale of the ORs (Varlog[OR]) than the tau^2^[IV] there was strong evidence of heterogeneity in meta-analyses comparing risk between Pacific Islander and White/European women in the US (95.2%) and New Zealand (100.0%).

### Publication bias assessment

For preterm birth prevalence meta-analyses, we did not identify any publication bias (Egger’s test, US: P = 0.54, USAPI: P = 0.35, New Zealand: P = 0.53, Papua New Guinea: P = 0.27). This assessment was not available for Australia since Egger’s test cannot be used with <3 studies. For the risk comparison meta-analyses, the robust Bayesian model expressed weak evidence of publication bias in the US (probability = 0.42, inclusion BF = 0.73) and New Zealand (probability = 0.48, inclusion BF = 0.93).

## Discussion

### Main findings

To our knowledge, this is the first systematic review and meta-analysis to summarize data from both Pacific Islander immigrants in high income settings and those resident in the Pacific Islands. Results indicate that US-resident Pacific Islanders had a relatively higher prevalence of preterm birth than in New Zealand and Australia and experienced poorer birth outcomes than White women, which was an inequity not observed in New Zealand. Estimates in other settings were limited by sparse data.

Our Pacific Islander-specific findings mirror the most recent country level estimates reported by the WHO. In 2014, among the three developed nations, the US had the highest overall preterm birth prevalence (9.6%, uncertainty interval [UI] 10.3%-14.0%), while the estimates in New Zealand (7.5%, UI 7.0%-9.8%) and Australia (8.6%, UI 6.9%-9.5%) were similar [82]. Notably, in our analysis we identified disparities between Pacific Islander and white women that were present in the US, but not in New Zealand. In New Zealand, while Pacific Islanders have become a minority group since European occupation, they make up a far greater proportion (24.6% [7]) of the population than in the US (0.4%) [83]. As such, supportive and culturally-based health policies have been prioritized in New Zealand to care for populations with high need (including Māori and Pacific Islanders) [84], such as Very Low Cost Access (VLCA), *Te Tiriti o Waitangi* (the Treaty of Waitangi) [85], which protects Māori resources, and *iwi* (tribally)-based primary care consistent with Māori values, attitudes, and aspirations [86, 87]. In contrast, in the US Pacific Islander women have reported limited health care access related to citizenship [16, 17], discrimination [19], and mistrust of health professionals [15–18]. Replicating New Zealand’s focus on primary health care, support for culturally-sensitive initiatives, and efforts to decrease structural racism [87–89] may be important if US disparities are to be addressed.

While Australia has among the largest absolute number of Pacific Islander migrants, they make up a small proportion (0.9%) of the population^12^, which likely explains the limited number of Pacific Islander-related studies reporting preterm birth. Australia has continuously supported seven surrounding Pacific Island countries to promote universal health coverage since 1995, including Fiji, Kiribati, Nauru, Samoa, Solomon Islands, Tonga and Vanuatu [90], but did not systematically record maternal ethnicity other than Caucasian and Indigenous (Aboriginal and Torres Strait Islander) in their own health system before 1998 [91]. Studies indicate that language barriers and inadequate health care access may increase the burden of adverse pregnancy outcomes among Pacific Islander migrants to Australia [66], but further data, with improved specificity in race/ethnicity reporting, is needed to better understand their perinatal outcomes.

Data from the Pacific Islands themselves were sparse and limited conclusions about the prevalence of preterm birth. While Pacific Islander women in the USAPI had a lower preterm birth estimate than the US, the wider prediction interval should be noted. All of the USAPIs are medically underserved [92] and, although prenatal/obstetric care is available [93, 94], coverage in different settings varies [94, 95] limiting opportunities for systematic data collection. Further data is sorely needed to adequately understand the needs of these territories, which are heavily dependent on US grant funding to sustain their health care systems.

Although Papua New Guinea is the largest of the Pacific Islands, with 8.9 million residents [96], studies were limited by design and response rate. For several reasons, the estimated prevalence may not accurately reflect the national burden of preterm birth. First, most of the included studies were from Madang Province, where much research is concentrated since the province is home to one of the country’s two medical schools and the Papua New Guinea Institute of Medical Research. The relatively higher prenatal care coverage (63%) in this setting [97, 98] compared to other regions may reduce maternal morbidity during or after pregnancy, meaning that estimates for preterm birth prevalence may be lower than the whole nation. Second, although malaria—a known risk factor for preterm birth—is endemic to Papua New Guinea (86% of cases in WHO Western Pacific Region were from Papua New Guinea [14]), most women in the included studies had either taken malaria prophylaxis before the recruitment or were cured before giving birth [68, 69, 71] (one study did not report malaria related information [70]). This also may not be the case for women residing in more rural and less medically served settings. Finally, in one of the largest samples of women among the Papua New Guinea studies [68], preterm birth was defined as less than 38 weeks, which may also have influenced the estimate.

### Strengths and Limitations

Limitations of our study should be noted. Beyond the USAPIs, Papua New Guinea and New Zealand there was little data on perinatal health from the remaining Pacific Islands. We identified only one study from Vanuatu [73] (PTB prevalence: 8.0%), and one study from the Solomon Islands in which the reported preterm birth prevalence was 23.8% in 2011–2013 [72] indicating that there may be broad heterogeneity in preterm birth prevalence across the region that was not captured here. Even within our studies, a large degree of heterogeneity was evident in prevalence meta-analyses for the USAPI, Australia, and Papua New Guinea. Potential explanations could include the lack of consistency in GA measurement methods, the limited publications, and quality of health care provided by local clinics. Similar heterogeneity in the risk comparison meta-analysis in the US may be from different perinatal outcomes among Pacific Islander subgroups, particularly the much higher risk and prevalence of preterm birth among Marshallese women; in New Zealand, the heterogeneity may be from different study designs, varied GA measurement methods (most were unclear), and the unavoidable heterogeneity for smaller meta-analyses [33]. The large proportion of included studies that did not clearly state the methods used to estimated GA also should be noted.

Our study does, however, have several notable strengths. We systematically searched articles reporting preterm birth outcomes among Pacific Islanders, including grey literature and comprehensively conducted backward and forward citation chaining. Second, we used tau for the heterogeneity assessment instead of I^2^ statistics, which have been questioned for their reliability especially for prevalence meta-analysis [31] and meta-analyses with a small number of included studies [33]. Additionally, to increase the accuracy of our risk comparison estimates, we used a Bayesian meta-analysis approach that performs better than frequentist meta-analysis approach when a small number of studies are included [35], and the corresponding robust Bayesian modelling method for publication bias assessment.

### Interpretation

Our findings have important public health implications. In developed nations like the US and Australia, an improved record of race/ethnicity information and ethnicity-specific analyses are the first steps to understanding the burden of adverse pregnancy outcomes among minority groups. It is vital to identify related, contextually relevant risk factors to inform future polices to decrease health disparities. Lessons may be learned from New Zealand’s culturally-sensitive approach. In the USAPI, Papua New Guinea, and the other Pacific nations not represented here, basic health care infrastructure improvements are likely needed before the true burden of preterm birth can be understood.

## Conclusion

Existing literature indicates that Pacific Islanders in the US had a higher prevalence of preterm birth than in other global settings and experienced health inequities. Learning from New Zealand’s culturally-sensitive approach to health care provision may provide a starting point for addressing disparities. Data from other Pacific settings is sparse, limiting conclusions about prevalence. More data is needed to understand the true burden of preterm birth in the Pacific region.

## Supporting information

S1 Checklist PRISMA 2020 checklist. (DOCX)

S1 Table Search strategy for systematic review of the literature on MEDLINE ALL (Ovid). (DOCX)

S2 Table Screening questions. (DOCX)

S3 Table Articles excluded due to overlapping data (n = 31). (DOCX)

S4 Table Risk of bias assessment for the preterm birth prevalence meta-analysis using the JBI checklist [26]. (DOCX)

S5 Table Risk of bias assessment for the risk comparison of preterm birth meta-analysis using the JBI checklist [27]. (DOCX)

S6 Table Summary for adjustment methods and confounding factors (risk comparison of preterm birth meta-analysis). (DOCX)

S1 Fig Forest plot of preterm birth prevalence subgroup analysis by Pacific Islander ethnicity in the US^a a^ Please note, the overall estimate presented here is not expected to match that in Fig 2A since these analyses are restricted to only a sub-sample of the overall study population. (TIF)

S2 Fig Forest plot of preterm birth prevalence subgroup analysis by Pacific Islander ethnicity in the New Zealand^a a^ Please note, the overall estimate presented here is not expected to match that in Fig 2C since these analyses are restricted to only a sub-sample of the overall study population. (TIF)

S3 Fig Forest plot of risk of preterm birth comparing Pacific Islander women to non-Hispanic White women subgroup by ethnicity in the US. (TIF)

S1 Appendix R code for meta-analyses. (DOCX)
