# Supplementary material for: Global prevalence of preterm birth among Pacific Islanders: A systematic review and meta-analysis
Source: PLOS Glob Public Health. 2023 Jun 14;3(6):e0001000. doi: 10.1371/journal.pgph.0001000 (PMC10266634; doi:10.1371/journal.pgph.0001000)
Supplement: S1 Table — (DOCX) [file pgph.0001000.s002.docx]

**S1 Table** Search strategy for systematic review of the literature on MEDLINE ALL (Ovid)

| **#** | **Search Terms** | **Results** |
| --- | --- | --- |
| 1 | exp premature birth/ or exp infant, premature/ or exp obstetric labor, premature/ or exp infant, low birth weight/ or exp tocolytic agents/ or exp fetal membranes, premature rupture/ or exp infant, premature, diseases/ or exp retinopathy of prematurity/ or exp respiratory distress syndrome, newborn/ or exp kangaroo-mother care method/ or exp enterocolitis, necrotizing/ or exp bronchopulmonary dysplasia/ or (delivery, obstetric/ and pregnancy outcome/) | 205654 |
| 2 | (((preterm or pre-term) adj2 (deliver* or birth* or labo* or syndrome* or infant* or neonate* or pregnan* or newborn* or born* or rupture* or retinopath* or retin* or bronch* or pulmon* or health*)) or prematur* or pre-matur* or ((short* or small*) adj1 gestation*) or PPROM).mp. | 250661 |
| 3 | 1 or 2 | 344045 |
| 4 | oceania/ or Australasia/ or exp pacific islands/ or exp oceanic ancestry group/ | 74011 |
| 5 | ((pacific adj2 (island* or wom#n or mother* or population* or infant* or newborn* or ancestr* or born* or neonate* or pregnan*)) or pasifika or pacifica or Melanesia* or Micronesia* or Polynesia* or Hawai* or "Hawai’i" or "ni’ihau" or niihau* or "kaua’I" or Kauai* or "o’ahu" or oahu* or "moloka’i" or Molokai* or "lana’i" or lanai* or "kaho’olawe" or Kahoolawe* or maui* or austral island* or "tupua’I island" or bass island* or Australasia* or Australia*-pacific or south sea island* or caroline island* or carolin* or Carolinian* or Chamorro* or chuuk* or cook island* or easter island* or fiji* or futun* or guam* or "i-kiribati" or Kiribati* or kosrae* or maori* or mariana island* or mariana* or marshall island* or marshall* or new Caledonia* or niue* or ni-vanuatu or Tuvalu* or Tahiti* or palau* or Nauru* or papua new guinea* or Papua* or Solomon island* or tonga* or Tokelau* or pitcairn* or pitcairn island* or pohnpei* or phoenix island* or rawaki island* or rapa nui* or saipan* or American samoa* or samoa* or New Zealand*).mp. | 160060 |
| 6 | 4 or 5 | 168316 |
| 7 | 3 and 6 | 2390 |
| 8 | exp animals/ not humans/ | 4914252 |
| 9 | 7 not 8 | 2150 |

Search date: Nov 15 2021
