# Supplementary material for: Global prevalence of preterm birth among Pacific Islanders: A systematic review and meta-analysis
Source: PLOS Glob Public Health. 2023 Jun 14;3(6):e0001000. doi: 10.1371/journal.pgph.0001000 (PMC10266634; doi:10.1371/journal.pgph.0001000)
Supplement: S2 Table — (DOCX) [file pgph.0001000.s003.docx]

**S2** **Table** Screening questions

| 1. Was the article or title-abstract published in the English language? |
| --- |
| 1. Does the article discuss preterm birth outcomes? |
| 1. Is the Pacific Islander race group discussed in the article? |
| 1. Does the article disaggregate preterm birth outcomes for Pacific Islanders rather than aggregating with other ethnic groups? (Exclude if aggregated) |
| 1. Is the article a conference abstract or a master thesis? (Exclude if yes) 2. Is the article a case report? (Exclude if yes) |
| 1. Is the article focused on outcomes after multiple birth? (Exclude if yes) 2. Does the article report preterm birth among women with specific medical condition only? (Exclude if yes) |
