# Supplementary material for: Global prevalence of preterm birth among Pacific Islanders: A systematic review and meta-analysis
Source: PLOS Glob Public Health. 2023 Jun 14;3(6):e0001000. doi: 10.1371/journal.pgph.0001000 (PMC10266634; doi:10.1371/journal.pgph.0001000)
Supplement: S3 Table — (DOCX) [file pgph.0001000.s004.docx]

**S3** **Table** Articles excluded due to overlapping data (n=31)

| **Study** | **Dataset**  **(Data collection year)** | **Reason for exclusion** |
| --- | --- | --- |
| ***US*** |  |  |
| Hamilton BE, Ventura SJ. Characteristics of births to single-and multiple-race women: California, Hawaii, Pennsylvania, Utah, and Washington, 2003. National vital statistics reports. 2007 May 3;55(15). | National Natality files (2003) | A smaller sample size compared to Wong et al, 2008^80^. |
| Ju AC, Heyman MB, Garber AK, Wojcicki JM. Maternal obesity and risk of preterm birth and low birthweight in Hawaii PRAMS, 2000–2011. Maternal and child health journal. 2018 Jun;22(6):893-902. | Hawaii Pregnancy Risk Assessment Monitoring System (2003) | Records could be covered by Hirai et al, 2013^45^. |
| Hayes D, Shor R, Pieron P, Roberson E, Fuddy L. “Premature Birth Fact Sheet” Honolulu, HI: Hawai’i Department of Health, Family Health Services Division; November 2010. | Hawaii Pregnancy Risk Assessment Monitoring System (2003) | Records could be covered by Hirai et al, 2013^45^. |
| Martin JA, Hamilton BE, Sutton PD, Ventura SJ, Menacker F, Munson ML. Births: final data for 2002. National vital statistics reports. 2003 Dec 17;52(10):1-13. | US continent, Natality Data Files (2002) | Records could be covered by Hirai et al, 2013^45^. |
| Pieron, P. P. M. (2010). Do preterm birth disparities exist in hawaii?(Order No. 3415920). Available from ProQuest Dissertations & Theses Global. (734857833). Retrieved from https://www.proquest.com/dissertations-theses/do-preterm-birth-disparities-exist-hawaii/docview/734857833/se-2?accountid=15172 | Hawaii, Vital Statistics Birth Certificate Database (2003-2008) | Records could be covered by Hirai et al, 2013^45^. |
| Kim S, Choi S, Chung-Do JJ, Fan VY. Comparing Birth Outcomes in Hawai ‘i between US-and Foreign-Born Women. Hawai'i Journal of Medicine & Public Health. 2018 Aug;77(8):188. | Hawaii Vital Statistic Natality Birth Data (2004) | Records could be covered by Hirai et al, 2013^45^. |
| Wright TE, Tam E. Disparate rates of persistent smoking and drug use during pregnancy of women of Hawaiian ancestry. Ethnicity & disease. 2010 Jan 1;20(1):215. | Hawaii, Pacific Research Center on Early Human Development Study (2007-2008) | Records could be covered by Hirai et al, 2013^45^. |
| Mattheus D, Shannon M, Lim E, Gandhi K. The Association Between Socio-demographic Factors, Dental Problems, and Preterm Labor for Pregnant Women Residing in Hawai ‘i. Hawai'i Journal of Medicine & Public Health. 2016 Aug;75(8):219. | Hawaii, Pregnancy Risk Assessment Monitoring System (2009-2011) | Overlapped with Hirai et al, 2013^45^. Excluded due to a smaller sample size. |
| Washington State Department of Health. Perinatal Indicators Report for Washington State - 2018 Data. https://doh.wa.gov/sites/default/files/legacy/Documents/Pubs//950-153_PerinatalIndicatorsforWashingtonResidents.pdf | Washington State, birth certificate data (2008, 2014-2018) | Overlapped with Martin et al, 2019^43^. Excluded due to a smaller sample size. |
| Tiwari R, Enquobahrie DA, Wander PL, Painter I, Souter V. A retrospective cohort study of race/ethnicity, pre-pregnancy weight, and pregnancy complications. The Journal of Maternal-Fetal & Neonatal Medicine. 2021 Jun 8:1-8. | Washington State, Foundation for Health Care Quality's Obstetrical Care Outcomes Assessment Program (2014-2018) | Overlapped with Martin et al, 2019^43^. Excluded due to a smaller sample size. |
| Baruffi G, Fuddy LJ, Onaka AT, Alexander GR, Mor JM. Temporal trends in maternal characteristics and pregnancy outcomes: their relevance to the provision of health services. Hawaii, 1979-1994. Hawaii Medical Journal. 1997;56(6). | Hawaii State, birth certificate (1979-1994) | Records could be covered by Crowell et al, 2007^47^. |
| Baruffi G, Kieffer EC, Alexander GR, Mor JM. Changing pregnancy outcomes of Samoan women in Hawaii. Paediatric and perinatal epidemiology. 1999 Jul 1;13(3):254-68. | Hawaii State, birth certificate (the same datafile used in Baruffi et al, 1997 above) | Records could be covered by Crowell et al, 2007^47^. |
| Kieffer EC, Mor JM, Alexander GR. The perinatal and infant health status of Native Hawaiians. American Journal of Public Health. 1994 Sep;84(9):1501-4. | Hawaii Vital Statistic Natality Birth Data (1983-1987) | Records could be covered by Crowell et al, 2007^47^. |
| Fuddy LJ, Prince CB, Tang MC. Perinatal substance use among high risk women in Hawaii: patterns and impact on pregnancy outcomes. Asian American and Pacific Islander Journal of Health. 2003 Dec 1;10(1):50-7. | Hawaii State, Perinatal Support Services program (1994-1995) | Overlapped with Crowell et al, 2007^47^. Excluded due to a smaller sample size. |
| Rao AK, Daniels K, El-Sayed YY, Moshesh MK, Caughey AB. Perinatal outcomes among Asian American and Pacific islander women. American Journal of Obstetrics and Gynecology. 2006 Sep 1;195(3):834-8. | California, hospital records (1998-2003) | Overlapped with Schempf et al, 2010^75^. Excluded due to a smaller sample size. |
| Delara RM, Madden E, Bryant AS. Short interpregnancy intervals and associated risk of preterm birth in Asians and Pacific Islanders. The Journal of Maternal-Fetal & Neonatal Medicine. 2018 Jul 18;31(14):1894-9. | California, birth records (1999-2005) | Overlapped with Schempf et al, 2010^75^. Excluded due to a smaller sample size. |
| Prince CB, Song L, Quadri N, Baker KK. Epidemiology of Low Birth Weight and Preterm Delivery, in Hawaii, 2000-2001. Californian Journal of Health Promotion. 2003 Dec 31;1(SI):83-90. | Hawaii State, Pregnancy Risk Assessment Monitoring System (2000-2001) | Overlapped with Mathews et al, 2003^79^. Excluded due to a smaller sample size. |
| Washington State Department of Health. Preterm Delivery for Singleton Births. https://doh.wa.gov/sites/default/files/legacy/Documents/Pubs//160-015-MCHDataRptPrenatalDeliv.pdf | Data from Washington State Department of Health (2004-2015) | No formal report. Additionally, data could be covered by other studies conducted in State of Washington. |
| Morisaki N, Kawachi I, Oken E, Fujiwara T. Social and anthropometric factors explaining racial/ethnical differences in birth weight in the United States. Scientific reports. 2017 Apr 21;7(1):1-8. | US continent, Natality Data Files (2009-2011) | Overlapped with Hirai et al, 2013^45^ and Hawaii State Department of Health et al, 2019^50^. Excluded due to a smaller sample size. |
| Karasek D, Baer RJ, McLemore MR, Bell AJ, Blebu BE, Casey JA, Coleman-Phox K, Costello JM, Felder JN, Flowers E, Fuchs JD. The association of COVID-19 infection in pregnancy with preterm birth: A retrospective cohort study in California. The Lancet Regional Health-Americas. 2021 Oct 1;2:100027. | California Vital Statistics birth certificate (2020-2021) | Overlapped with Hamilton et al, 2021^46^ and Hamilton et al, 2022^53^. Excluded due to a smaller sample size. |
| ***New Zealand*** |  |  |
| Schellenberg JC, North RA, Taylor R, Zhou RL. Secretory component of immunoglobulin A in maternal serum and the prediction of preterm delivery. American journal of obstetrics and gynecology. 1998 Mar 1;178(3):535-9. | Birth records from National Women's Hospital, Middlemore Hospital, and North Shore Hospital (1993-1995) | Sample size was not large enough based on the JBI checklist. |
| Sadler L, Saftlas A, Wang W, Exeter M, Whittaker J, McCowan L. Treatment for cervical intraepithelial neoplasia and risk of preterm delivery. Jama. 2004 May 5;291(17):2100-6. | National Women's Hospital (1988-2000) | Sadler et al, 2004^61^ has a larger sample size. |
| Gao W, Paterson J, Carter S, Percival T. Risk factors for preterm and small‐for‐gestational‐age babies: A cohort from the Pacific Islands Families Study. Journal of paediatrics and child health. 2006 Dec;42(12):785-92. | Middlemore Hospital (2000) | A smaller sample size compared to Sundborn et al, 2011^60^. |
| Edmonds LK, Sibanda N, Geller S, Cram F, Robson B, Filoche S, Storey F, Gibson‐Helm M, Lawton B. He Tamariki Kokoti Tau: Tackling preterm incidence and outcomes of preterm births by ethnicity in Aotearoa New Zealand 2010–2014. International Journal of Gynecology & Obstetrics. 2021 Nov;155(2):239-46. | National Minimum Dataset (2010-2014) | Study duration is covered in Berry et al, 2018^64^. |
| Ministry of Health New Zealand Government. Report on Maternity 2014. https://www.birthbythenumbers.org/wp-content/uploads/2016/06/NZReport-on-maternity-2014.pdf | National Minimum Dataset (2010-2014) | Study duration is covered in Berry et al, 2018^64^. |
| Thayer Z, Bécares L, Atatoa Carr P. Maternal experiences of ethnic discrimination and subsequent birth outcomes in Aotearoa New Zealand. BMC Public Health. 2019 Dec;19(1):1-8. | North Island of Aotearoa,  Growing Up in New Zealand Study (2009-2010) | Study duration is covered in Berry et al, 2018^64^. |
| Gurney J, Sarfati D, Stanley J, Studd R. Do ethnic patterns in cryptorchidism reflect those found in testicular cancer?. The Journal of urology. 2013 Nov 1;190(5):1852-7. | Maternity Data Collection (2000-2010) | A smaller sample size compared to Berry et al, 2018^64^. Some overlaps on time duration. |
| ***Australia*** |  |  |
| Farrant BM, Shepherd CC. Maternal ethnicity, stillbirth and neonatal death risk in Western Australia 1998–2010. Australian and New Zealand Journal of Obstetrics and Gynaecology. 2016 Oct;56(5):532-6. | Data Linkage Branch of the Western Australian Government Department of Health (1998-2010) | A smaller sample size compared to Mozooni et al, 2018^66^. |
| ***Papua New Guinea*** |  |  |
| O'Donnell A, Raiko A, Clegg JB, Weatherall DJ, Allen SJ. α+‐Thalassaemia and pregnancy in a malaria endemic region of Papua New Guinea. British journal of haematology. 2006 Oct;135(2):235-41. | Claimed in the article that they used the same dataset with Allen et al, 1998^66^. | A smaller sample size compared to Allen et al, 1998^68^. |
| Ome-Kaius M, Unger HW, Singirok D, Wangnapi RA, Hanieh S, Umbers AJ, Elizah J, Siba P, Mueller I, Rogerson SJ. Determining effects of areca (betel) nut chewing in a prospective cohort of pregnant women in Madang Province, Papua New Guinea. BMC Pregnancy and childbirth. 2015 Dec;15(1):1-9. | Hospital records in Madang Province (2009-2012) | Unger et al, 2019^71^ covered a longer study duration. |
| Unger HW, Ome-Kaius M, Wangnapi RA, Umbers AJ, Hanieh S, Suen CS, Robinson LJ, Rosanas-Urgell A, Wapling J, Lufele E, Kongs C. Sulphadoxine-pyrimethamine plus azithromycin for the prevention of low birthweight in Papua New Guinea: a randomised controlled trial. BMC medicine. 2015 Dec;13(1):1-6. | Hospital records in Madang Province (2009-2013) | Unger et al, 2019^71^ covered a longer study duration. |
| Fowkes FJ, Moore KA, Opi DH, Simpson JA, Langham F, Stanisic DI, Ura A, King CL, Siba PM, Mueller I, Rogerson SJ. Iron deficiency during pregnancy is associated with a reduced risk of adverse birth outcomes in a malaria-endemic area in a longitudinal cohort study. BMC medicine. 2018 Dec;16(1):1-0. | Hospital records in Madang Province (2005-2007) | A smaller sample size compared to Senn et al, 2009^68^. |
