# Supplementary material for: Global prevalence of preterm birth among Pacific Islanders: A systematic review and meta-analysis
Source: PLOS Glob Public Health. 2023 Jun 14;3(6):e0001000. doi: 10.1371/journal.pgph.0001000 (PMC10266634; doi:10.1371/journal.pgph.0001000)
Supplement: S4 Table — (DOCX) [file pgph.0001000.s005.docx]

**S4** **Table** Risk of bias assessment for the preterm birth prevalence meta-analysis using the JBI checklist^26^

| **Study (Data collection year)** | **JBI quality assessment checklist for prevalence studies** | | | | | | | | | **Total Score %** |
| --- | --- | --- | --- | --- | --- | --- | --- | --- | --- | --- |
|  | **Q1** | **Q2** | **Q3** | **Q4** | **Q5** | **Q6** | **Q7** | **Q8** | **Q9** |  |
| ***US (N=17, included studies for subgroup analyses)*** | | | | | | | | | | |
| Crowell et al., 2007 (1968-1994)^47^ | Y | Y | Y | Y | Y | Y | Y | Y | Y | 100.0 |
| Andrasfay et al., 2021 (1978-1995)^48^ | Y | Y | Y | Y | Y | Y | Y | Y | U | 88.9 |
| Korinek et al., 2021 (1989-2015)^44^ | Y | Y | Y | Y | Y | Y | U | Y | U | 77.8 |
| Nembhard et al., 2019 (1997-2013)^42^ | Y | Y | Y | Y | Y | Y | Y | Y | Y | 100.0 |
| Mathews et al., 2003 (2001)^79^ | Y | Y | Y | Y | Y | Y | Y | Y | Y | 100.0 |
| Hirai et al., 2013 (2002-2009)^45^ | Y | Y | Y | Y | Y | Y | Y | Y | Y | 100.0 |
| Centers for Disease Control and Prevention et al., 2011 (2003-2008)^52^ | Y | Y | Y | Y | Y | Y | U | Y | U | 77.8 |
| Wong et al., 2008 (2003)^80^ | Y | Y | Y | Y | Y | Y | U | Y | U | 77.8 |
| Schempf et al., 2010 (2003-2005)^75^ | Y | Y | Y | Y | Y | Y | Y | Y | Y | 100.0 |
| Altman et al., 2019 (2007-2012)^49^ | Y | Y | Y | Y | Y | Y | U | Y | Y | 88.9 |
| Wartko et al., 2017 (2008-2012)^41^ | Y | Y | Y | Y | Y | N | U | Y | Y | 77.8 |
| Hawaii State Department of Health et al., 2019 (2012-2015)^50^ | Y | Y | Y | Y | Y | Y | Y | Y | N | 88.9 |
| Public Health Department, Seattle & King County et al., 2015 (2013)^51^ | Y | Y | Y | Y | Y | Y | U | Y | U | 77.8 |
| Quan et al., 2021 (2015-2017)^81^ | Y | Y | Y | Y | Y | Y | Y | Y | U | 88.9 |
| Martin et al., 2019 (2016-2018)^43^ | Y | Y | Y | Y | Y | Y | Y | Y | U | 88.9 |
| Hamilton et al., 2021 (2019-2020)^46^ | Y | Y | Y | Y | Y | Y | Y | Y | U | 88.9 |
| Hamilton et al., 2022 (2021)^53^ | Y | Y | Y | Y | Y | Y | Y | Y | U | 88.9 |
| ***USAPI (N=5)*** | | | | | | | | | | |
| Fox et al., 2005 (1986-1996)^58^ | Y | Y | Y | Y | Y | Y | U | Y | U | 77.8 |
| DelaCruz et al., 2018 (2007-2014)^57^ | Y | Y | Y | Y | Y | Y | Y | Y | Y | 100.0 |
| Berger et al., 2016 (2007-2013)^56^ | Y | Y | Y | Y | Y | Y | Y | Y | N | 88.9 |
| Ministry of Health in Republic of the Marshall Islands et al., 2010 (2007-2010)^55^ | Y | Y | Y | Y | Y | Y | U | Y | U | 77.8 |
| Ministry of Health in Republic of the Marshall Islands et al., 2016 (2011-2016)^54^ | Y | Y | Y | Y | Y | N | U | Y | U | 66.7 |
| ***New Zealand (N=7)*** | | | | | | | | | | |
| Cantwell et al., 1973 (Not reported)^63^ | Y | U | Y | Y | U | N | U | Y | Y | 55.6 |
| Wright et al., 1998 (1987-1990)^59^ | Y | Y | Y | Y | Y | Y | Y | Y | U | 88.9 |
| Sadler et al., 2002 (1992-1999)^61^ | Y | Y | Y | Y | Y | Y | U | Y | U | 77.8 |
| Lawton et al., 2016 (1995-2009)^62^ | Y | Y | Y | Y | Y | Y | U | Y | Y | 88.9 |
| Sundborn et al., 2011 (2000)^60^ | Y | Y | Y | Y | Y | Y | U | Y | Y | 88.9 |
| Berry et al., 2018 (2005-2015)^64^ | Y | Y | Y | Y | Y | Y | U | Y | Y | 88.9 |
| Ministry of Health et al., 2019 (2016)^65^ | Y | Y | Y | Y | Y | Y | U | Y | U | 77.8 |
| ***Australia (N=2)*** | | | | | | | | | | |
| Berman et al., 2021 (2003-2016)^67^ | Y | Y | Y | Y | Y | Y | U | Y | Y | 88.9 |
| Mozooni et al., 2018 (2005-2013)^66^ | Y | Y | Y | Y | Y | Y | U | Y | U | 77.8 |
| ***Papua New Guinea (N=4)*** | | | | | | | | | | |
| Garner et al., 1994 (1984-1987)^69^ | Y | Y | Y | Y | Y | Y | Y | Y | U | 88.9 |
| Allen et al., 1998 (1994-1996)^68^ | Y | Y | Y | Y | Y | Y | Y | Y | U | 88.9 |
| Senn et al., 2009 (2007-2008)^70^ | Y | Y | Y | Y | Y | Y | U | Y | U | 77.8 |
| Unger et al., 2019 (2009-2013)^71^ | Y | Y | Y | Y | Y | Y | Y | Y | U | 88.9 |

Annotation:

Y – Yes; N – No; U – Unclear; NA – Not applicable.

Q1: Was the sample frame appropriate to address the target population?

Q2: Were study participants sampled in an appropriate way?

Q3: Was the sample size adequate?

Q4: Were the study subjects and the setting described in detail?

Q5: Was the data analysis conducted with sufficient coverage of the identified sample?

Q6: Were valid methods used for the identification of the condition?

Q7: Was the condition measured in a standard, reliable way for all participants?

Q8: Was there appropriate statistical analysis?

Q9: Was the response rate adequate, and if not, was the low response rate managed appropriately?
