# Supplementary material for: Global prevalence of preterm birth among Pacific Islanders: A systematic review and meta-analysis
Source: PLOS Glob Public Health. 2023 Jun 14;3(6):e0001000. doi: 10.1371/journal.pgph.0001000 (PMC10266634; doi:10.1371/journal.pgph.0001000)
Supplement: S5 Table — (DOCX) [file pgph.0001000.s006.docx]

**S5** **Table** Risk of bias assessment for the risk comparison of preterm birth meta-analysis using the JBI checklist^27^

| **Study (Data collection year)** | **JBI quality assessment checklist for analytical cross-sectional studies** | | | | | | | | **Total Score %** |
| --- | --- | --- | --- | --- | --- | --- | --- | --- | --- |
|  | **Q1** | **Q2** | **Q3** | **Q4** | **Q5** | **Q6** | **Q7** | **Q8** |  |
| ***US (N=12, included studies for subgroup analyses)*** | | | | | | | | | |
| Crowell et al., 2007 (1968-1994)^47^ | Y | Y | Y | Y | NA | NA | Y | NA | 62.5 |
| Andrasfay et al., 2021 (1989-2015)^48^ | Y | Y | Y | Y | NA | NA | Y | NA | 62.5 |
| Korinek et al., 2021 (1989-2015)^44^ | Y | Y | Y | Y | Y | Y | U | Y | 87.5 |
| Nembhard et al., 2019 (1997-2013)^42^ | Y | Y | Y | Y | Y | Y | Y | Y | 100 |
| Hirai et al., 2013 (2002-2009)^45^ | Y | Y | Y | Y | NA | NA | Y | NA | 62.5 |
| Schempf et al., 2010 (2003-2005)^75^ | Y | Y | Y | Y | Y | Y | Y | Y | 100 |
| Ratnasiri et al., 2018 (2007-2016)^74^ | Y | Y | Y | Y | Y | Y | Y | Y | 100 |
| Wartko et al., 2017 (2008-2012)^41^ | Y | Y | Y | N | NA | NA | U | NA | 37.5 |
| Hawaii State Department of Health et al., 2019 (2012-2015)^50^ | Y | Y | Y | Y | NA | NA | Y | NA | 62.5 |
| Public Health Department, Seattle & King County et al., 2015 (2013)^51^ | N | Y | Y | Y | NA | NA | U | NA | 37.5 |
| Martin et al., 2019 (2016-2018)^43^ | Y | Y | Y | Y | NA | NA | Y | NA | 62.5 |
| Hamilton et al., 2021 (2019-2020)^46^ | N | Y | Y | Y | NA | NA | Y | NA | 50 |
| Hamilton et al., 2022 (2021)^53^ | N | Y | Y | Y | NA | NA | Y | NA | 50 |
| ***New Zealand (N=3)*** | | | | | | | | | |
| Craig et al., 2004 (1996-2001)^76^ | Y | Y | Y | Y | Y | Y | Y | Y | 100.0 |
| Parry et al., 2011 (2007-2010)^78^ | Y | Y | Y | Y | NA | NA | Y | NA | 62.5 |
| Edmonds et al., 2021 (2010-2014)^77^ | Y | Y | Y | Y | NA | NA | U | NA | 50 |

Annotation:

Y – Yes; N – No; U – Unclear; NA – Not applicable.

Q1: Were the criteria for inclusion in the sample clearly defined?

Q2: Were the study subjects and the setting described in detail?

Q3: Was the exposure measured in a valid and reliable way?

Q4: Were objective, standard criteria used for measurement of the condition?

Q5: Were confounding factors identified?

Q6: Were strategies to deal with confounding factors stated?

Q7: Were the outcomes measured in a valid and reliable way?

Q8: Was appropriate statistical analysis used?
