# Supplementary material for: Global prevalence of preterm birth among Pacific Islanders: A systematic review and meta-analysis
Source: PLOS Glob Public Health. 2023 Jun 14;3(6):e0001000. doi: 10.1371/journal.pgph.0001000 (PMC10266634; doi:10.1371/journal.pgph.0001000)
Supplement: S6 Table — (DOCX) [file pgph.0001000.s007.docx]

**S6** **Table** Summary for adjustment methods and confounding factors (risk comparison of preterm birth meta-analysis)

| **Study**  **(Data collection)** | **Method of adjustment** | **Measure of association** | **Confounding factors** |
| --- | --- | --- | --- |
| ***US (N=12)*** | | | |
| Crowell et al., 2007 (1968-1994)^47^ | None | OR^a^ | None. |
| Andrasfay et al., 2021 (1989-2015)^48^ | None | OR^a^ | None. |
| Korinek et al., 2021 (1989-2015)^44^ | Logistic regression | OR^b^ | Mother's national origin, mother ethnicity, year of birth, mother's age, mother's marital status, mother's educational status. |
| Nembhard et al., 2019 (1997-2013)^42^ | Modified Poisson regression | RR^c^ | Maternal age, education, parity, and marital status. |
| Hirai et al., 2013 (2002-2009)^45^ | None | OR^a^ | None. |
| Schempf et al., 2010 (2003-2005)^75^ | Logistic regression | OR | Maternal nativity, age, education, marital status, parity, prenatal care, tobacco use, and state of residence. |
| Ratnasiri et al., 2018 (2007-2016)^74^ | Logistic regression | OR | Birth year, maternal age, race/ethnicity, maternal education, maternal nativity, maternal demographic region, source of prenatal care payment, WIC participation, first trimester prenatal care initiation, parity, maternal smoking, maternal pre-pregnancy BMI. |
| Wartko et al., 2017 (2008-2012)^41^ | None | OR^a^ | None. |
| Hawaii State Department of Health et al., 2019 (2012-2015)^50^ | None | OR^a^ | None. |
| Public Health Department, Seattle & King County et al., 2015 (2013)^51^ | None | OR^a^ | None. |
| Martin et al., 2019 (2016-2018)^43^ | None | OR^a^ | None. |
| Hamilton et al., 2021 (2019-2020)^46^ | None | OR^a^ | None. |
| Hamilton et al., 2022 (2021)^53^ | None | OR^a^ | None. |
| ***New Zealand (N=3)*** | | | |
| Craig et al., 2004 (1996-2001)^76^ | Logistic regression | OR | Maternal age, NZDep Index decile, year, age*year. |
| Parry et al., 2011 (2007-2010)^78^ | None | OR^a^ | None. |
| Edmonds et al., 2021 (2010-2014)^77^ | None | OR^a^ | None. |

BMI, body mass index; NZDep Index decile, New Zealand Index of Deprivation.

^a^ The association estimate was calculated from the prevalence among two race populations.

^b^ The association estimate was calculated from the coefficient estimates.

^c^ Transferred into OR for the meta-analysis estimate.
