# Supplementary figures and images for: Global prevalence of preterm birth among Pacific Islanders: A systematic review and meta-analysis
Source: PLOS Glob Public Health. 2023 Jun 14;3(6):e0001000. doi: 10.1371/journal.pgph.0001000 (PMC10266634; doi:10.1371/journal.pgph.0001000)

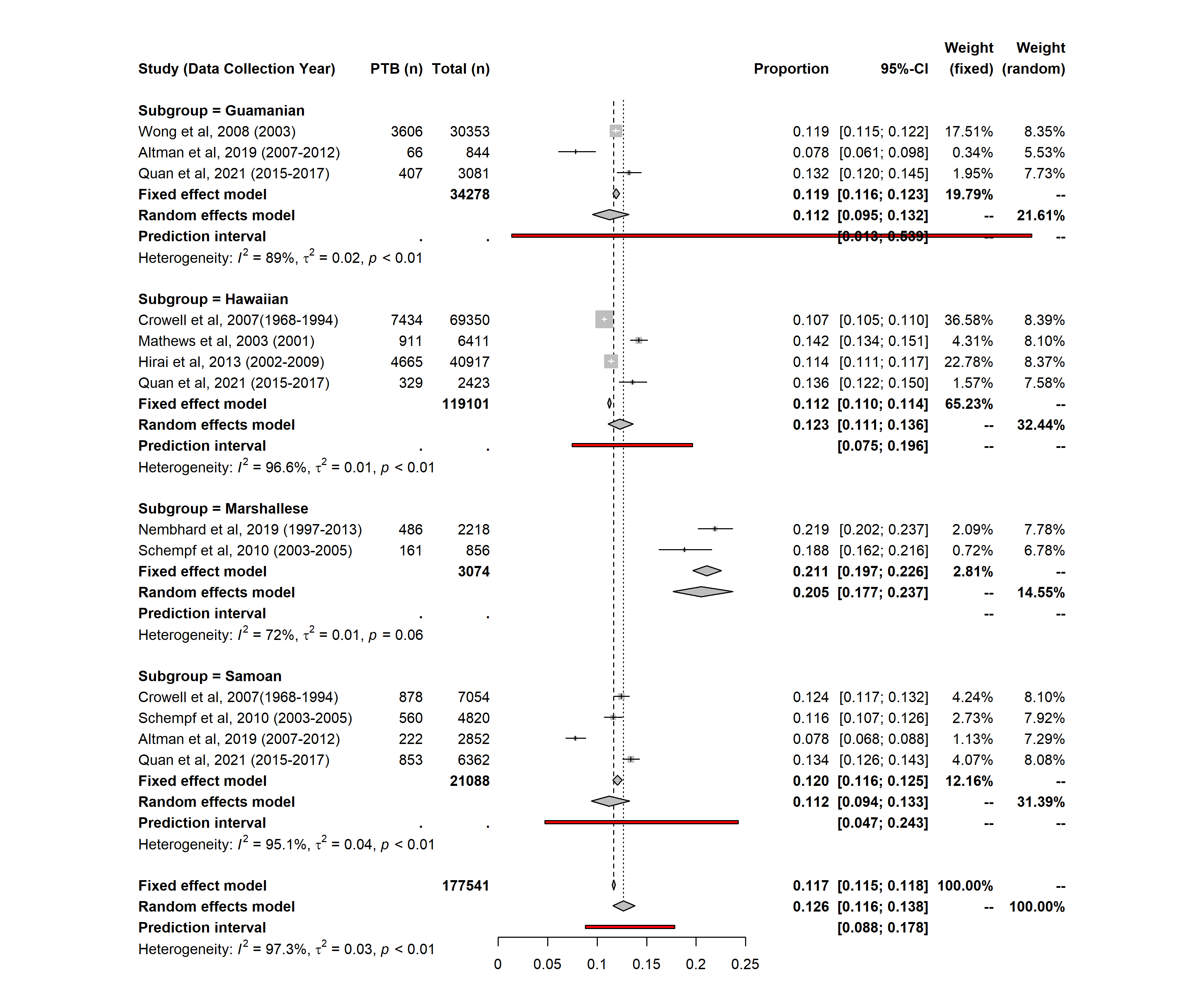

Supplement: S1 Fig — (TIF) [file pgph.0001000.s008.tif]

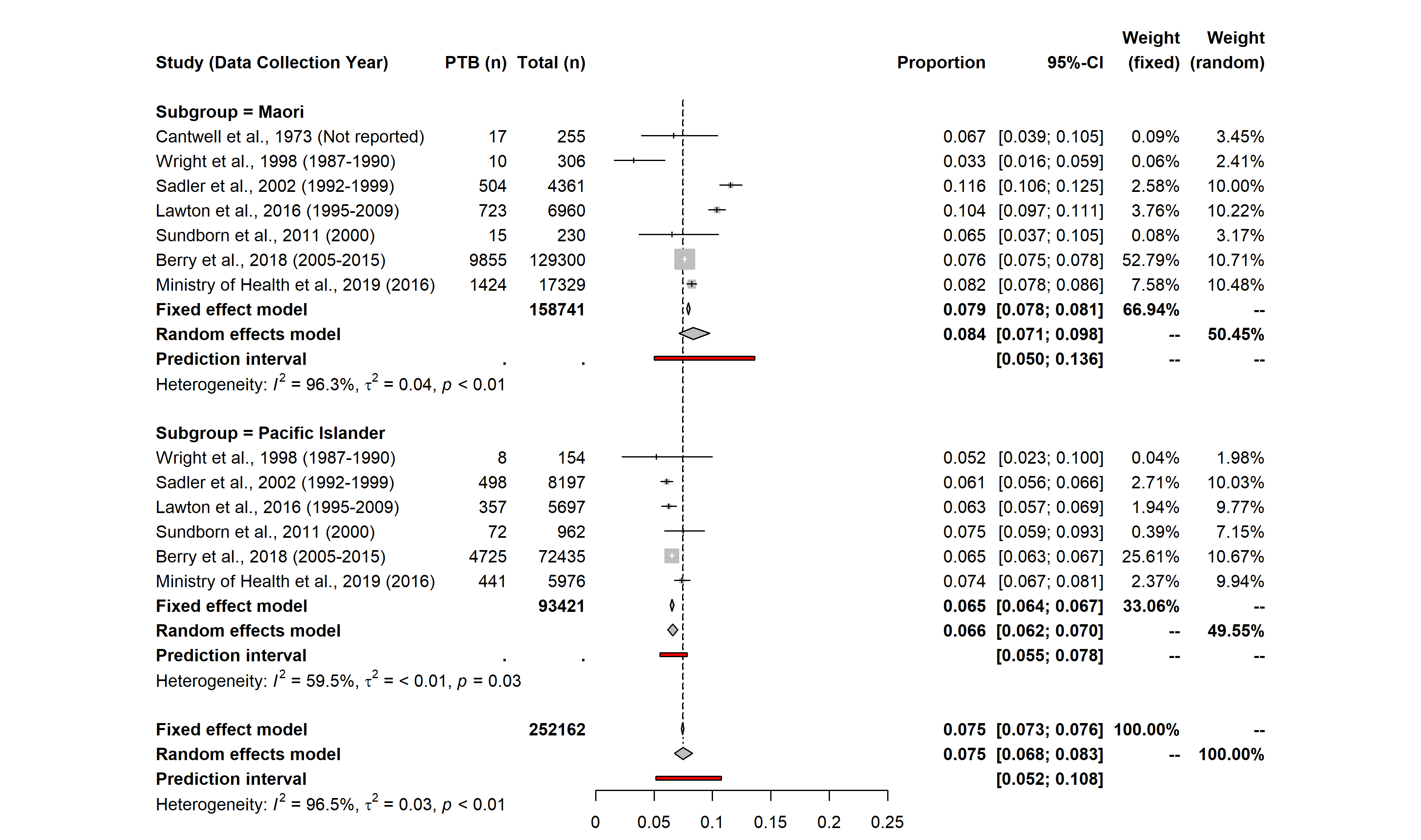

Supplement: S2 Fig — (TIF) [file pgph.0001000.s009.tif]

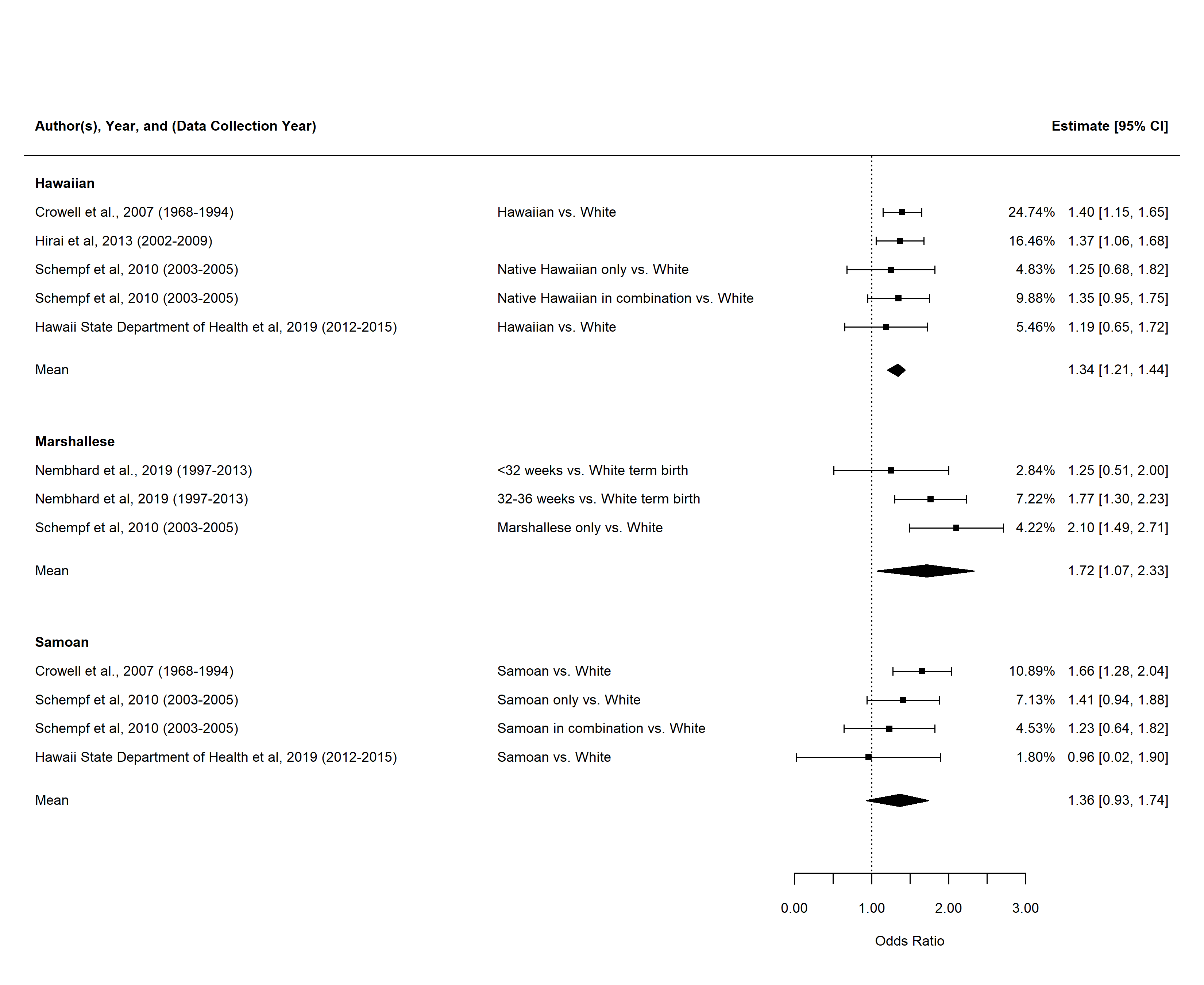

Supplement: S3 Fig — (TIF) [file pgph.0001000.s010.tif]
