# Supplementary material for: Global prevalence of preterm birth among Pacific Islanders: A systematic review and meta-analysis
Source: PLOS Glob Public Health. 2023 Jun 14;3(6):e0001000. doi: 10.1371/journal.pgph.0001000 (PMC10266634; doi:10.1371/journal.pgph.0001000)
Supplement: S1 Appendix — (DOCX) [file pgph.0001000.s011.docx]

**S8** **Appendix** R code for meta-analyses

################################################################################

################### R code for PLOS Global Public Health manuscript ####################

######################### Using the US datasets as an example ########################

################################################################################

#### Install packages ####

install.packages("tidyverse")

install.packages("meta")

install.packages("metafor")

devtools::install_github("MathiasHarrer/dmetar")

install.packages("bayesmeta")

install.packages("RoBMA") # requires R 4.0.0

#### Load libraries into R ####

library(meta)

library(dmetar)

library(metafor)

library(bayesmeta)

library(RoBMA)

#### Preterm Birth prevalence meta-analysis in the US ####

### Importing from .csv file

### us.prevalence = PTB prevalence data among Pacific Islanders in the US

us.prevalence <- read.csv("<insert path to file>\\us.prevalence.csv")

### Description for variables in the dataset us.prevalence

### event = Number of PTB cases

### total = sample size of the population

### author_year = Label of included studies

### race_ethnicity = Subgroup ethnicity information

### Forestplot

meta.us.prevalence <- metaprop(event=event, n=total, studlab=author_year,

data=us.prevalence,method="Inverse",

method.tau="DL",prediction=T)

forest(meta.us.prevalence,digits = 3, digits.se=2, digits.tau2 = 2,

digits.weight = 2,comb.fixed = T, digits.I2 = 1, leftlabs = c("Study","PTB (n)","Total (n)"),

col.square = "gray", col.diamond = "gray", col.predict = "red",

fs.study = 10, fs.study.labels = 10, fs.heading = 10, fs.random = 10,

fs.hetstat = 10, fs.axis = 10, fs.smlab = 10, fs.fixed = 10)

### tau square statistics

metaprop(event=event, n=total, studlab=author_year, data=us.prevalence,

method="Inverse", method.tau="DL")

### publication bias

eggers.test(x=us.prevalence)

### subgroup analysis

meta.us.prevalence.sub <- metaprop(event=event, n=total, studlab=author_year,

byvar=droplevels(race_ethnicity), data=us.prevalence)

forest(meta.us.prevalence.sub,digits = 3, digits.se=2, digits.tau2 = 2,

digits.weight = 2,comb.fixed = F, digits.I2 = 1, leftlabs = c("Study","PTB (n)","Total (n)"),

col.square = "gray", col.diamond = "gray", fs.study = 10, fs.study.labels = 10, fs.heading = 10,

fs.random = 10, fs.hetstat = 10, fs.axis = 10, fs.smlab = 10, col.by="black", bylab = "Subgroup")

#### Preterm Birth risk comparison between Pacific Islander and white women in the US ####

### Importing from .csv file

### us.or = Risk of PTB comparing Pacific Islander women to white women in the US

us.or <- read.csv("<insert path to file>\\us.or.csv")

### Description for variables in the dataset us.prevalence

### or = odds ratio of PTB comparing Pacific Islander women to white women

### or_lnse = log standard error of the odds ratio

### author_year = Label of included studies

### race_ethnicity = Subgroup ethnicity information

### Forestplot

meta.us.or <- escalc(measure = "OR", yi=or, sei=or_lnse,slab=author_year, data=us.or)

meta.us.or.b <- bayesmeta(y = meta.us.or[, "yi"], sigma = sqrt(meta.us.or[, "vi"]),

labels = meta.us.or[,"author_year"], mu.prior.mean = 0, mu.prior.sd = 4,

tau.prior = function(t) dhalfnormal(t, scale = 0.5))

forest(meta.us.or.b, digits=2, cex=0.75, xlab = "Odds Ratio", xlim=c(-8,5),

cex.lab=0.75, cex.axis = 0.75, refline = 1.47, header=c("Author(s), Year, and (Data Collection Year)"),

annotate=T, showweights=T)

### subgroup analysis

ha.idx <- us.or[us.or$ethnicity=="Hawaiian",]

ma.idx <- us.or[us.or$ethnicity=="Marshallese",]

sa.idx <- us.or[us.or$ethnicity=="Samoan",]

meta.us.ha <- escalc(measure = "OR", yi=or, sei=or_lnse,slab=author_year, data=us.or[ha.idx,])

meta.us.ha.b <- bayesmeta(y = meta.us.ha[, "yi"], sigma = sqrt(meta.us.ha[, "vi"]),

labels = meta.us.ha[,"author_year"], mu.prior.mean = 0, mu.prior.sd = 4,

tau.prior = function(t) dhalfnormal(t, scale = 0.5))

meta.us.ma <- escalc(measure = "OR", yi=or, sei=or_lnse,slab=author_year, data=us.or[ma.idx,])

meta.us.ma.b <- bayesmeta(y = meta.us.ma[, "yi"], sigma = sqrt(meta.us.ma[, "vi"]),

labels = meta.us.ma[,"author_year"], mu.prior.mean = 0, mu.prior.sd = 4,

tau.prior = function(t) dhalfnormal(t, scale = 0.5))

meta.us.sa <- escalc(measure = "OR", yi=or, sei=or_lnse,slab=author_year, data=us.or[sa.idx,])

meta.us.sa.b <- bayesmeta(y = meta.us.sa[, "yi"], sigma = sqrt(meta.us.sa[, "vi"]),

labels = meta.us.sa[,"author_year"], mu.prior.mean = 0, mu.prior.sd = 4,

tau.prior = function(t) dhalfnormal(t, scale = 0.5))

forest(meta.us.or.b, digits=2, cex=0.75, xlab = "Odds Ratio", xlim=c(-8,5),

cex.lab=0.75, cex.axis = 0.75, refline = 1.36, rows=c(1:4, 9:11, 16:20),

header=c("Author(s), Year, and (Data Collection Year)"), annotate=T, showweights=T)

## add subgroup outcomes

addpoly(x=1.343, ci.lb=1.207, ci.ub=1.438, row=14.5, cex=0.75, mlab = c("Mean")) # Hawaiian

addpoly(x=1.715, ci.lb=1.069, ci.ub=2.331, row=7.5, cex=0.75, mlab = c("Mean")) # Marshallese

addpoly(x=1.364, ci.lb=0.930, ci.ub=1.742, row=-0.5, cex=0.75, mlab = c("Mean")) # Samoan

## add text

par(font=2,cex=0.75)

text(-10, c(5,12,21), pos=4, c("Samoan", "Marshallese", "Hawaiian"))

par(font=1,cex=0.75)

text(-4, c(20:16, 11:9, 4:1), pos=4, us.or$race_ethnicity)

### publication bias assessment

fit.us <- RoBMA(y=meta.us.or.b$y , se=meta.us.or.b$sigma,

study_names = as.character(meta.us.or.b$author_year)
